# Supplementary material for: Demonstration of non-destructive and isotope-sensitive material analysis using a short-pulsed laser-driven epi-thermal neutron source
Source: Nat Commun. 2022 Mar 4;13:1173. doi: 10.1038/s41467-022-28756-0 (PMC8897477; doi:10.1038/s41467-022-28756-0)
Supplement: Supplementary file 1 — Supplementary Information [file 41467_2022_28756_MOESM1_ESM.pdf]

SUPPLEMENTARY MATERIAL

NRS evaluation

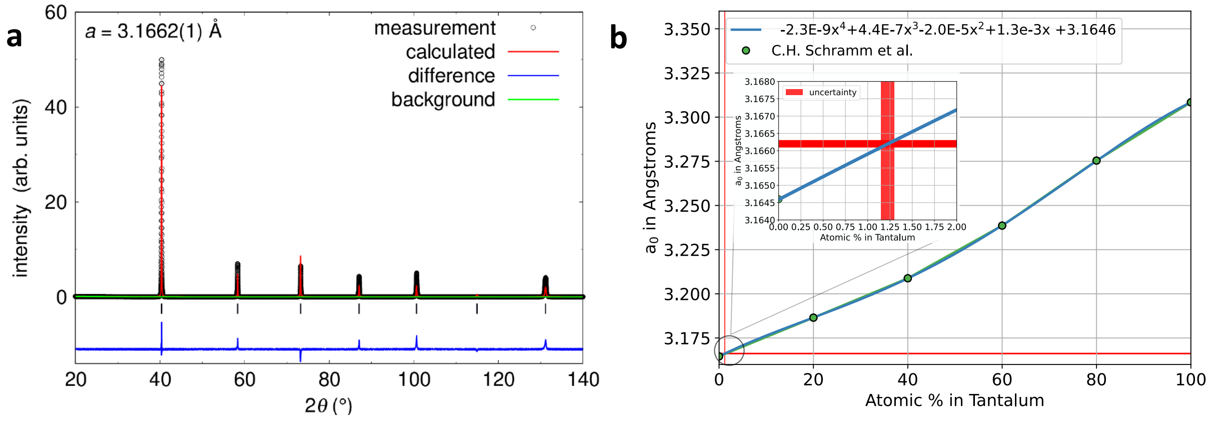

Supplementary Fig. 1. **Determination of the tantalum content. a:** The lattice parameter a_0 of the tungsten sample was investigated with X-ray diffraction. a_0 was determined to 3.1662±0.0001 angstrom. **b:** Schramm et al. [2] has found that a_0 changes with the percentage of tantalum present in tungsten (green dots). A 4th order polynomial fit was used to interpolate between the data points. Based on these findings the tantalum impurity has been determined to be at 1.23±0.07%. The red area identifies the range of uncertainty for the measurement.

The energy resolution of the setup is influenced by the intrinsic energy uncertainty caused by the moderation process. For the ^{182}W resonance, this leads to an energy uncertainty ΔE_M=0.12 eV. The Doppler broadening for this energy is ΔE_D=0.05 eV. Additional contributions to the uncertainty are caused by the distance (± 1 cm $\widehat{=}$ 0.05 eV) as well as the data processing (0.07 eV). This leads to a total energy uncertainty of 0.16 eV for this resonance. The presence of the tantalum impurity was investigated with energy dispersive spectroscopy on a scanning electron microscope and with X-ray diffraction. The measurement of the lattice parameter was a_0=3.1662(1) Å which corresponds to an atomic percentage of 1.23±0.07 % tantalum [2] which is close to the detection limit. By applying equation 5 to the ^{181}Ta resonance at 10 eV, it is possible to estimate that the minimal atomic presence of tantalum in the sample has to be higher

that 0.6 % to be still detectable. These ranges are deemed to be realistic for the industrial grade tungsten sample. The results of a laser-driven neutron activation analysis is attached separately and further confirms the presence of tantalum.

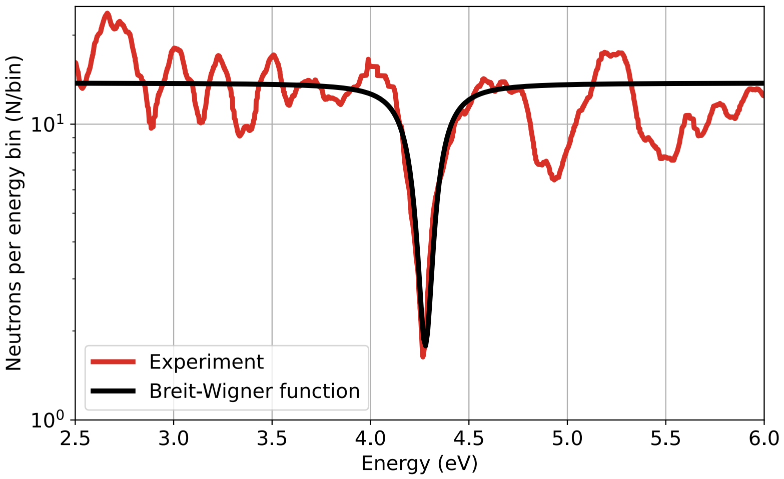

Supplementary Fig. 2. **Determination of the resonance position.** A small bin size with additional smoothing is used to reduce fluctuations caused by the binning position and enables a more precise determination of the resonance position. The center of the resonance is determined with a Breit-Wigner fit to 4.28 eV. It is worth noting that the resonance width Γ= 0.18 eV is smaller than the simulated value Γ= 0.55 eV.

In Fig. 2.**c** of the publication, the resonance shape was evaluated with a large bin with to reduce the statistical fluctuations per bin. This resulted in an energy uncertainty of 0.4 eV due to the large binning. This resolution can be increased in the evaluation algorithm by using a smaller binning and apply a smoothing function. This distorts the shape of the resonance but enables a more accurate determination of the resonance position. In Supplementary Fig. 2, the binning is set to a average width of $E_n/85$ which enables a determination of the resonance position of 4.28 eV by a fit to the Breit-Wigner function. This is within the calculated energy uncertainty to the literature value of 4.15 eV [3]. It has to be noted that the width of the resonance is reduced

by this process to Γ=0.18 eV. A pure tungsten sample with a natural ^{182}W content of 26.5 % [4] would result in a FWHM of 0.55 eV according to Monte Carlo simulations. The measured resonance width that is smaller than the literature value is probably due to a lack of statistics and can be considered an artefact.

SUPPLEMENTARY REFERENCES

[1] DB Syme, "The black and white filter method for background determination in neutron time-of-flight spectrometry," Nuclear Instruments and Methods in Physics Research **198**, 357–364 (1982).

[2] CH Schramm, P Gordon, and AR Kaufmann, "The alloy systems uranium-tungsten, uranium-tantalum and tungsten-tantalum," Jom **2**, 195–204 (1950).

[3] MB Chadwick, P Obložinskỳ, Michal Herman, NM Greene, RD McKnight, DL Smith, PG Young, RE MacFarlane, GM Hale, SC Frankle, *et al.*, "Endf/b-vii. 0: next generation evaluated nuclear data library for nuclear science and technology," Nuclear data sheets **107**, 2931–3060 (2006).

[4] John R De Laeter, John Karl Böhlke, Paul De Bièvre, Hiroshi Hidaka, H Steffen Peiser, Kevin JR Rosman, and Philip DP Taylor, "Atomic weights of the elements. review 2000 (iupac technical report)," Pure and applied chemistry **75**, 683–800 (2003).

[5] T Ziegler, C Bernert, S Bock, F-E Brack, TE Cowan, NP Dover, M Garten, L Gaus, R Gebhardt, U Helbig, *et al.*, "PW-class laser-driven proton acceleration optimization by application of temporally asymmetric pulse shapes," arXiv preprint arXiv:2007.11499 (2020).

[6] Marc Zimmer, *Laser-Driven Neutron Sources-A Compact Approach to Non-Destructive Material Analysis*, Ph.D. thesis, Technische Universität Darmstadt (2020).
