# Supplementary figures and images for: Demonstration of non-destructive and isotope-sensitive material analysis using a short-pulsed laser-driven epi-thermal neutron source
Source: Nat Commun. 2022 Mar 4;13:1173. doi: 10.1038/s41467-022-28756-0 (PMC8897477; doi:10.1038/s41467-022-28756-0)

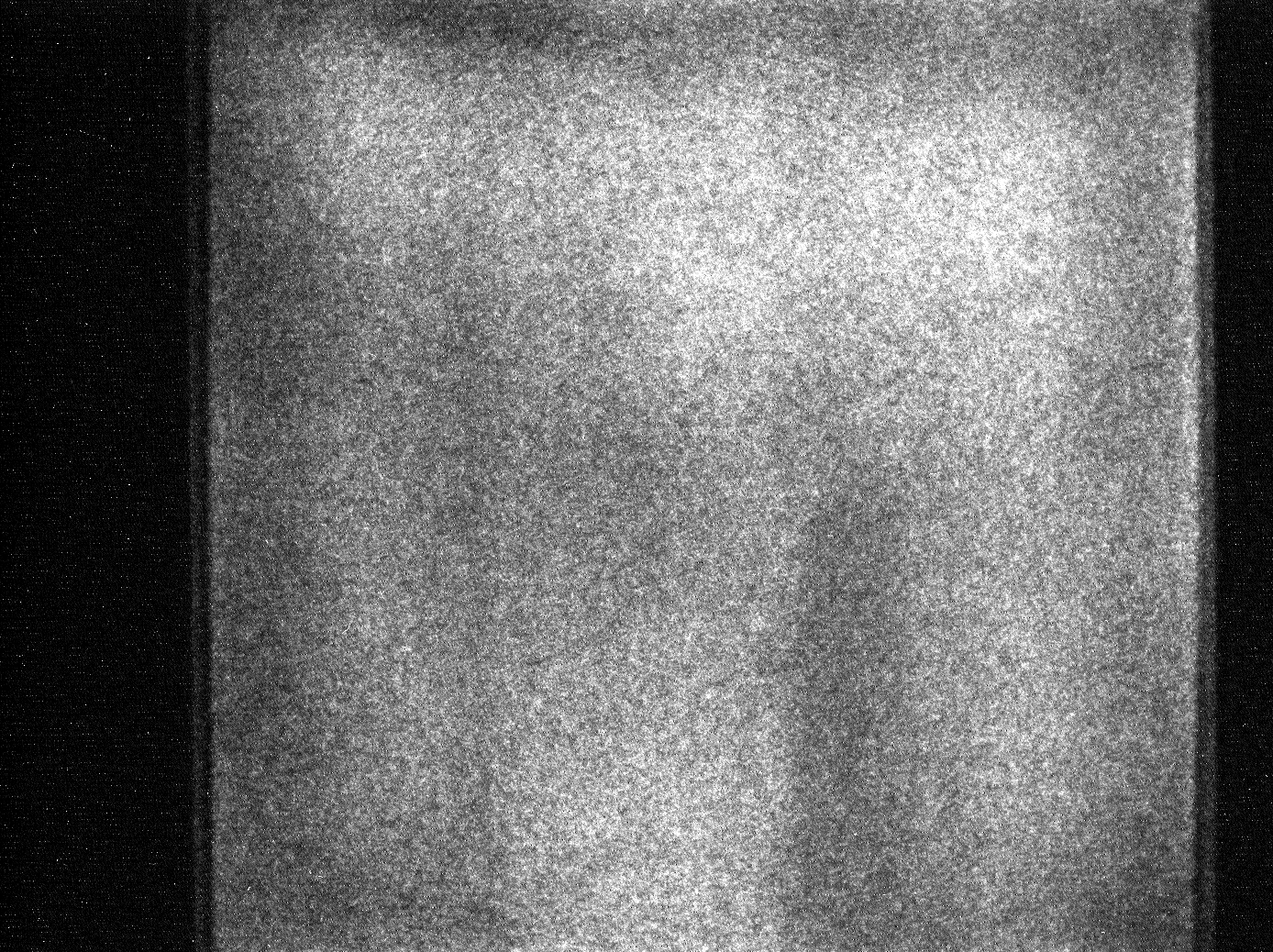

Supplement: Supplementary file 3 — Source Data [file 41467_2022_28756_MOESM3_ESM.zip › Data/Neutron Radiography/Background.tiff]

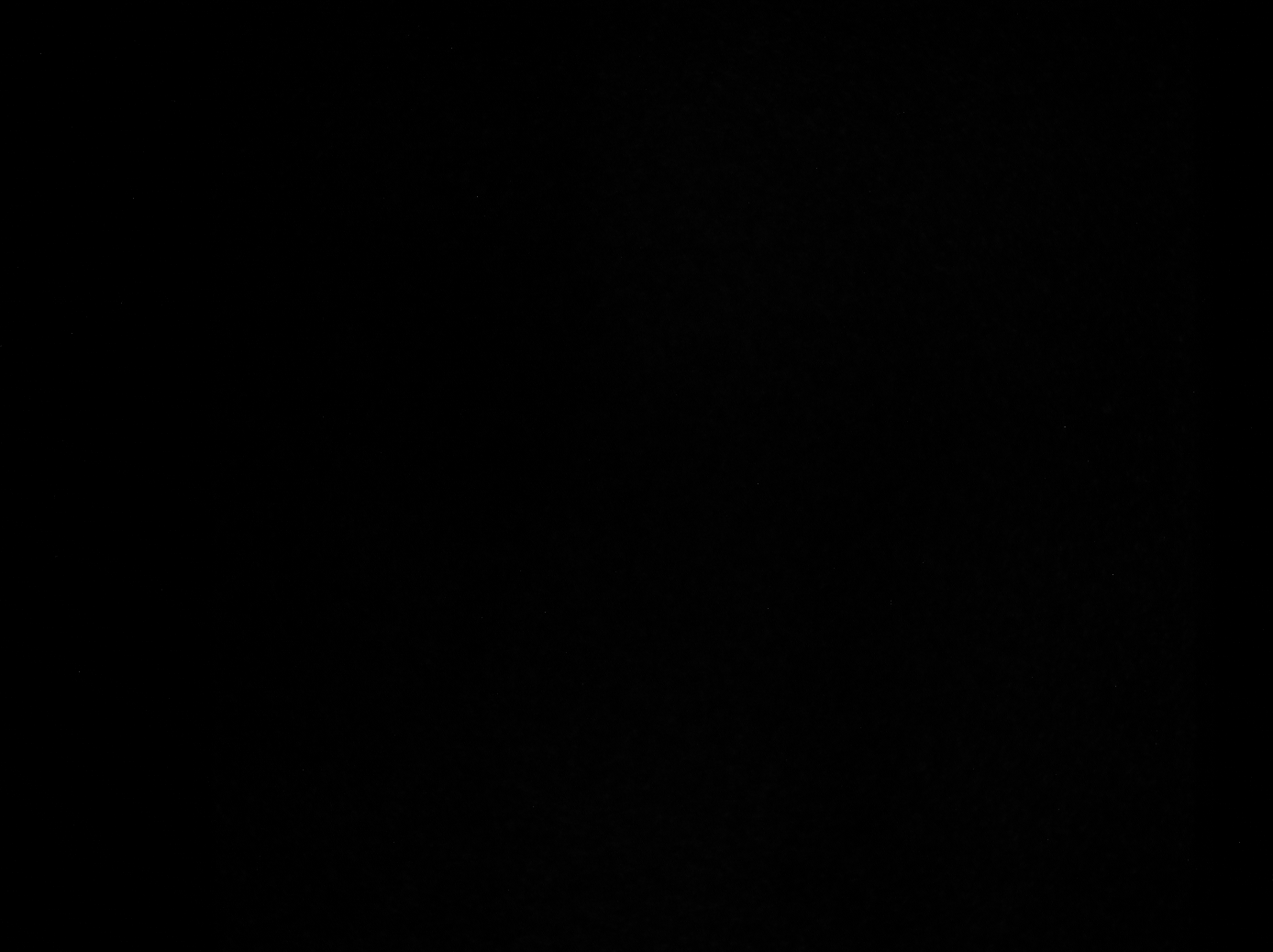

Supplement: Supplementary file 3 — Source Data [file 41467_2022_28756_MOESM3_ESM.zip › Data/Neutron Radiography/Singleshot.tiff]
